# The Care of the Sick in Prisons

**Published:** 1896-03-28

**Authors:** F. M. F. Skene


### The Care of the Sick in Prisons.

By F. M. F. Skene.

The remarkable improvement which this century has witnessed in the organisation of prisons and the general treatment of criminals has been strikingly manifested, most especially in the regulations which now obtain in all our gaols and convict establishments for the care of the sick. This fact is probably well known to all who have been allowed to inspect any of our large prisons under a temporary order of admission, and who have thus learnt as much of their internal economy as can be known in a few hours' visit; but there are peculiar difficulties connected with the duties of a medical prison officer, which can only be rightly estimated by those who are in a position to study constantly the details of that silent life which is hidden from all eyes behind impenetrable walls. The arrangements made for dealing with illness of every description in the hospital department may now be pronounced simply admirable. In large prisons, such as Wormwood Scrubs, where upwards of a thousand prisoners have to be dealt with day by day, there are separate wards for the different forms of disease which may manifest themselves—a ward for infectious cases, kept totally apart from the rest of the prison; a lying-in ward, a convalescent ward, and one for more serious illness, and, besides these, four "sick cells," as they are called, for the care of slighter ailments. The wards are large, well ventilated, warmed with hot-water pipes, having a separate kitchen and bath-room attached to them, and furnished with every appliance necessary for the satisfactory treatment of the patients. There are also padded cells where the insane or violent prisoners can be placed in safety. Skilled officers—male and female, respectively—are placed in charge of the hospital department, two, if necessary, in each ward by night, and three by day, as their exclusive range of duty, and their attention and kindness to the sick is, as a rule, beyond praise. In smaller prisons, where there is neither space nor necessity for such extensive arrangements, there is one large infirmary ward on the male side, and another of similar extent for the use of the female prisoners; while in both divisions places of reception for infectious cases are provided, generally in what are called "combination cells," that is, two large cells thrown into one. Apart from these arrangements for the treatment of actual illness among the prisoners, their general health is attended to with the utmost care and regularity. The medical officer arrives at an early hour every morning—in large prisons he is required to be present twice daily; the warders bring before him every prisoner who is supposed to be ailing even in the slightest degree, and he deals carefully with each case, ordering modification of the rigid diet rules, or relaxation of labour, &c., as he may think necessary. The most exemplary precautions are taken where there is the least suspicion of mental disease, or when the prisoner is subject to epileptic fits. All such are at once placed "under observation," which phrase implies that they are never left alone, but are watched night and day by competent persons. As may be supposed, there are often cases of delirium tremens among those newly incarcerated, and we regret that restrictions of space prevent us from describing the remarkable improvement in health often experienced by prisoners under long sentences, and which is mainly due to their enforced sobriety and regular habits of life, in spite of their somewhat meagre diet, for though they live on wholesome well-cooked food, it is decidedly restricted in quantity. We have said that there are peculiar difficulties in the work of a prison medical officer, and they are to some extent due to the fact that the plans for the care of the sick are so excellent. It will readily be understood that to be promoted to the infirmary, excused from labour, and provided with an agreeable diet, seems, especially to the long-sentence prisoner, the entrance into a species of Elysium—they are ready to welcome real and even painful maladies which would produce this happy result for them, but where an unfortunate course of stolid good health gives no hope of a consummation so devoutly to be wished, they have recourse to deception. The extraordinary devices to which they resort for this purpose could hardly be believed unless actually witnessed. They often inflict positive tortures upon themselves in order to simulate serious disease or a grievous hurt; knocking the head violently against the wall is a favourite plan. A man has also been known to lie for very many hours refraining from the slightest movement even of the eyelids, and enduring without flinching all sorts of sharp expedients undertaken to prove that he is not really unconscious as he wishes to be supposed. It would, however, take too long to describe all the rough treatment to which they submit their bodies with this view, and the doctor is generally too wise and experienced to be deceived, but there is one expedient much patronised by them which renders a few days in hospital really necessary. They can always hang themselves, in so nicely calculated a manner as to ensure their being cut down before life is absolutely extinct. While everything that could possibly facilitate self-destruction is carefully kept from them, and even their clothes are removed from their cells at night, they cannot be altogether deprived of the means of strangulation, for which the possession of a shirt easily torn into strips would suffice. The officers make their systematic rounds of the cells with such unfailing precision that the hospital aspirant can time his supposed demise so as to be certainly found hanging, while it is still possible to restore animation. A singular instance occurred not long since where the pretended suicide overreached himself and became one in reality. His cell was at one end of a row extending along both sides of a wide corridor, and, according as the officer inspecting them in the morning made his round from right to left, or *vice versâ*, his cell would be the first or the last to be visited. It had always been the custom of the officer to begin at the right side, which would have brought him to the suicide's cell in excellent time to have him cut down alive, but it happened—unknown, of course, to the prisoner—that a new officer had been appointed for this duty, who found it more convenient to begin his inspection on the left. It took a considerable time, with the result that when he arrived at the last cell its crafty inmate was dead. Various circumstances proved afterwards that he had not had the smallest intention of killing himself. It will be seen, even from these brief notes, that the medical officer of a prison ought to possess not only skill, but very marked gifts of penetration and tact.

